# A next-generation sequencing method for gene doping detection that distinguishes low levels of plasmid DNA against a background of genomic DNA

**DOI:** 10.1038/s41434-019-0091-6

**Published:** 2019-07-11

**Authors:** Eddy N. de Boer, Petra E. van der Wouden, Lennart F. Johansson, Cleo C. van Diemen, Hidde J. Haisma

**Affiliations:** 1 University of Groningen, University Medical Center Groningen, Department of Genetics, Groningen, the Netherlands; 2 University of Groningen, Department of Chemical and Pharmaceutical Biology, Groningen Research Institute of Pharmacy, Groningen, the Netherlands

**Keywords:** Genetic techniques, Genetic vectors, Genetic vectors

## Abstract

Gene doping confers health risks for athletes and is a threat to fair competition in sports. Therefore the anti-doping community has given attention on its detection. Previously published polymerase chain reaction-based methodologies for gene doping detection are targeting exon–exon junctions in the intron-less transgene. However, because these junctions are known, it would be relatively easy to evade detection by tampering with the copyDNA sequences. We have developed a targeted next-generation sequencing based assay for the detection of all exon–exon junctions of the potential doping genes, *EPO*, *IGF1*, *IGF2*, *GH1,* and *GH2*, which is resistant to tampering. Using this assay, all exon–exon junctions of copyDNA of doping genes could be detected with a sensitivity of 1296 copyDNA copies in 1000 ng of genomic DNA. In addition, promotor regions and plasmid-derived sequences are readily detectable in our sequence data. While we show the reliability of our method for a selection of genes, expanding the panel to detect other genes would be straightforward. As we were able to detect plasmid-derived sequences, we expect that genes with manipulated junctions, promotor regions, and plasmid or virus-derived sequences will also be readily detected.

## Introduction

Doping is a threat to the integrity of sport and the health of athletes. Although there is no current evidence that gene doping has ever been used, continuous improvements in gene-therapy techniques increase the likelihood of abuse. Therefore, since 2004, the anti-doping community has been given attention on developing a test for the detection of gene doping [1, 2].

Gene doping refers to the hypothetical nontherapeutic use of gene-therapy by athletes to improve their performance. Although one can only speculate about the manner of administration, the most likely method would be injection of transgenes into the skeletal muscle in the form of viral constructs, after which the biochemical machinery of the cell would be recruited to express the transgene [3–5]. The most reliable assay to detect this form of gene doping would require a muscle biopsy, but such an invasive procedure is not appropriate [4, 5]. However, in this scenario, small amounts of transgenes will leak into the bloodstream, and these can be isolated from a huge excess of genomic DNA (gDNA). As gene doping would most likely use copyDNA instead of gDNA to reduce the size of the transgene, polymerase chain reaction (PCR) methods have been developed for the detection of copyDNA from in-vivo-administered genes in blood, proving the presence of transgenes in blood [3, 6–8].

Currently published methods for detection of gene doping use PCR-based methods or loop-mediated isothermal amplification (LAMP) that target unique sequences in a doping gene corresponding to exon–exon junctions in the intron-less transgene [3, 5–13]. However, because the exon–exon junctions of doping genes are known and the short PCR primers are even interrupted by the slightest change of the sequence, it is relatively simple to evade detection using current PCR-based methods by modifying the doping gene with for example synonymous mutations, which will then give a false-negative result.

**Fig. 1:**
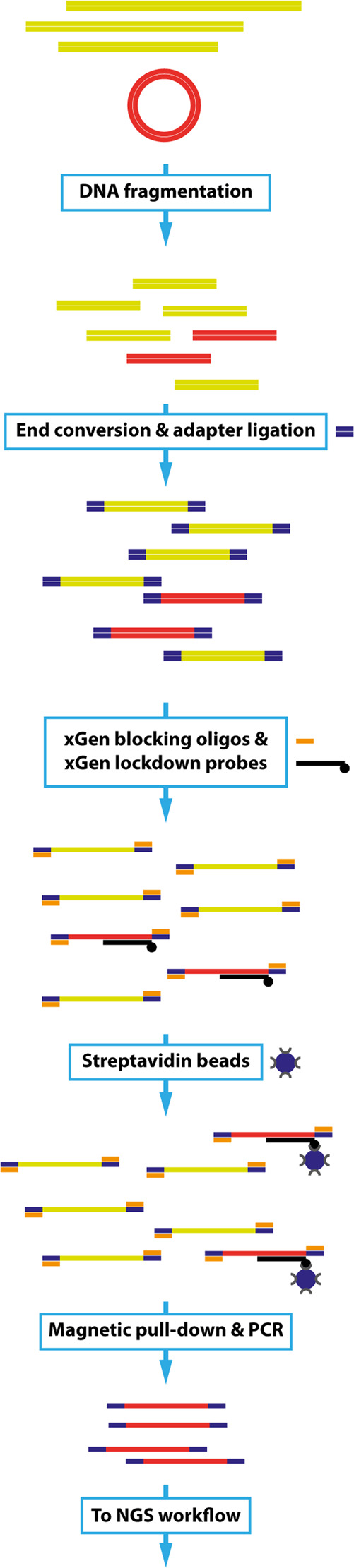
Schematic overview of the NGS gene doping detection assay. Genomic DNA with potential traces of gene doping copyDNA in the form of plasmids is isolated from blood. Isolated DNA is fragmented and the fragments are prepared for the sequence procedure by adding sequence adapters. Gene doping copyDNA fragments are hybridized to biotin-labeled xGen lockdown probes targeted to all exon–exon junctions of all known gene doping transcripts. xGen blocking oligos are added during hybridization to prevent nonspecific binding of the xGen lockdown probes to the sequence adapters. After hybridization, the captured fragments are magnetically pulled down with streptavidin beads, PCR-amplified and sequenced on an Illumina MiSeq sequencer

Here we describe a new gene doping detection assay that overcomes this problem. The test is based on targeted next-generation sequencing (NGS) of the copyDNA of potential doping genes that targets all exon–exon junctions of all transcripts of these genes (Fig. 1). Our method is currently set up for the reliable routine detection of the potential doping genes *EPO*, *IGF1*, *IGF2*, *GH1*, and *GH2*, but it is not restricted to these genes.

## Materials and methods

### Plasmids and copyDNA samples

Real-life gene doping blood samples will consist of a mixture of a high percentage of gDNA (>99%) and a low percentage of copyDNA (<1%) from the doping gene in the form of copyDNA in either plasmids or viruses. We imitated this composition by mixing commercially available plasmids containing the copyDNA sequence of potential doping genes and high molecular gDNA (>60 kilobases (kb)) from a pool of donors. For quantification purposes, a standard nonhuman gene-coding plasmid, pEGFP-N1 (*EGFP*), was chosen. The plasmids pcDNA3.1+/C-(K)DYK-EPO, pcDNA3.1+/C-(K)DYK-GH1, pcDNA3.1+/C-(K)DYK-GH2, pcDNA3.1+/C-(K)DYK-IGF1 and pcDNA3.1+/C-(K)DYK-IGF2 [Genescript, Piscataway Township, NJ, USA] encoding erythropoietin (*EPO*), growth hormone 1 (*GH1*), growth hormone 2 (*GH2*), insulin like growth factor 1 (*IGH1*) and insulin like growth factor 2 (*IGH2*) were used for validation of the method (Supplementary information 1).

### Dilution series

CopyDNA concentrations were measured using the Qubit dsDNA HS Assay [Thermo Fisher Scientific, Waltham, MA, USA]. gDNA concentration was measured using spectrophotometry [Nanodrop, Thermo Fisher Scientific]. CopyDNA and gDNA were mixed at equimolar ratios assuming a gDNA molecular weight equivalent to 80 kb. To determine the sensitivity and specificity of our method, we prepared dilutions containing one or multiple gene doping copyDNA samples: **1:** 1% *EPO*, 1% *EGFP*, 98% gDNA; **2:** 0.1% *EPO*, 1% *EGFP*, 98.9% gDNA; **3:** 0.01% *EPO*, 1% *EGFP*, 98.99% gDNA; **4:** 1% *GH1*, 1% *EGFP*, 98% gDNA; **5:** 0.1% *GH1*, 1% *EGFP*, 98.9% gDNA; **6:** 0.01% *GH1*, 1% *EGFP*, 98.99% gDNA; **7:** 0.1% all doping genes (0.1% *EPO*, 0.1% *GH1*, 0.1% *GH2*, 0.1% *IGF1*, 0.1% *IGF2*, 1% *EGFP*, 98.5% gDNA) and **8:** 0.01% all doping genes (0.01% *EPO*, 0.01% *GH1*, 0.01% *GH2*, 0.01% *IGF1*, 0.01% *IGF2*, 1% *EGFP*, 98.95% gDNA). A total of 2.6 µg DNA per sample was mixed in 130 µl Tris-EDTA.

### Calculating the number of copies copyDNA per 1000 ng gDNA

*EPO* 0.01% is used as an example for this calculation. The calculation is based on the assumption that the average weight of a base pair (bp) is 650 Daltons, meaning that the molecular weight of a 6044 bp plasmid is 3,928,600 g per mole. The inverse of the molecular weight, the plasmid concentration, is 2.545 × 10^−7^ mole/g. Using Avogadro’s number (6.022 × 10^23^ molecules/mole) the number of plasmids per gram is 1.533 × 10^17^ copies. We added 0.00846 × 10^−9^ g plasmid to 1000 ng gDNA, which is equivalent to 1,296,800 plasmid copies.

### Library preparation of samples

DNA of the dilution series was fragmented by sonication using Covaris Sonalab 7.1 S220 [Covaris, Woburn, MA, USA] (80 s, peak power 140.0, duty factor 10.0, cycles/burst 200, power ~ 12, temp below 12 °C). Shearing results were checked by electrophoresis using an Agilent D1000 screen tape [Agilent, Santa Clara, CA, USA]. The mean size of the fragmented DNA was ~300 bp. Sample preparation was performed using the NEBNext Ultra II DNA Library Prep Kit for Illumina sequencing [New England Biolabs, Ipswich, MA, USA] using an input amount of 1 µg DNA in 50 µl Tris-EDTA. NEB adapters were substituted for unique molecular identifier (UMI) TruSeq dual-index duplex adapters (15 µM) [Integrated DNA Technologies (IDT), Coralville, IA, USA], and USER enzyme steps were skipped. UMIs are used to remove duplicate reads and reduce the error rate during the data-analysis procedure. A size-selection to 300–400 bp using AMPure XP Beads [Beckman Coulter, Indianapolis, IN, USA] was performed after adding adapters. IDT xGen Library Amplification primers (5 µM p5 and 5 µM p7) were used to enrich the adapter-ligated DNA using PCR (12 cycles). The amplified product was measured by electrophoresis using the Agilent High Sensitivity D1000 screen tape after cleanup with AMPure XP beads.

### Design of capturing probes

One hundred and twenty base pair sequences of all protein-coding exon–exon junctions of *EPO*, *GH1*, *GH2*, *IGF1,* and *IGF2* transcripts were collected in a FASTA file using ENSEMBL GRCh37 and GRCh38 (www.ensembl.org) [European Molecular Biology Laboratory’s European Bio-informatics Institute, Hinxton, UK]. Care was taken to have the exon–exon junction in the middle of the probe-sequence. Overlapping and complementary sequences were prevented by choosing the complementary strand if necessary. Plasmid *EGFP*, sized the median transcript length of the regions of interest, was added for quantification purposes. IDT designed biotin-labeled probes to the regions in the FASTA file using the xGen LockDown probes protocol (Supplementary information 2). The *EGFP* sequence was fully tiled with 120 bp biotin-labeled probes. The quality of each synthesized probe was individually determined by chromatography, mass spectrophotometry and electrospray ionization. Quality performance of the probes was checked using the basic local alignment search tool (BLAST) (https://blast.ncbi.nlm.nih.gov/) [National Center for Biotechnology Information, Rockville Pike, Bethesda, MD, USA] and measurement of GC-percentage. Detailed information about the xGen LockDown probes protocol is available from IDT upon request.

### Enrichment procedure

5′-biotinylated xGen lockdown probes [IDT] were used to enrich the region of interest following the manufacturer’s instructions (hybridization capture of DNA libraries using xGen lockdown probes and reagents). In short, 300 ng of each sample-prepped library was 8-plexed and dried using a vacuum concentrator at a maximum of 70 °C [Speedvac, Thermo Fisher Scientific]. Probes were hybridized to their target, and the hybridized library was captured with M-270 streptavidin Dynabeads [Thermo Fisher Scientific]. xGen Library Amplification primers were used to enrich the captured library (13 cycles). The amplified product was measured by electrophoresis using the Agilent High Sensitivity D1000 screen tape after cleanup with AMPure XP beads.

### Sequencing

The sequence procedure was performed on an Illumina MiSeq sequencer [Illumina, San Diego, CA, USA] (V2, 2 × 150 bp reads) following the manufacturer’s instructions. FASTQ files for index reads in MiSeq Reporter were generated according to Illumina instructions.

### Data-analysis

Demultiplexing was done automatically by MiSeq Reporter using the unique sample-barcodes. Data analysis started with the demultiplexed reads that passed filter stored in zipped FASTQ files and contained the automated steps: (1). Unzip FASTQ file. 1b. Reads optionally pre-aligned to the human reference genome (human_g1k_v37) using BWA MEM [14], leaving the unmapped sequence reads for further processing. To prevent copyDNA reads from being mapped, we changed the band width to 10 bp in the pre-alignment. (2). Extract UMI sequences, for all or unmapped sequences, from the index read and put all reads in an unmapped BAM file using fgbio v5.0.1 FastqToBam (https://github.com/fulcrumgenomics/fgbio/releases). (3). Convert unmapped BAM file to FASTQ file using Picard v2.10.0 (https://broadinstitute.github.io/picard/) [Broad Institute, Cambridge, MA, USA] SamToFastq. (4). Map FASTQ file to reference FASTA files of interest using BWA MEM -p -t 8. The plasmid *EGFP* FASTA file contains the plasmid sequence. The gene doping FASTA files contain the coding sequence of a specific transcript. (5). Sort unmapped BAM files by query name using Picard SortSam. (6). Receive UMI information from the unmapped BAM files to the mapped BAM files using Picard MergeBamAlignment (SO = coordinate, ALIGNER_PROPER_PAIR_FLAGS = true, MAX_GAPS = −1, ORIENTATIONS = FR, VALIDATION_STRINGENCY = SILENT, CREATE_INDEX = true). (7). Group mapped reads by UMI using fgbio GroupReadsByUmi (strategy = adjacency). (8). Create consensus reads based on UMIs using fgbio CallMolecularConsensusReads (error-rate-post-umi = 30, min-reads = 1). (9). Convert BAM reads to FASTQ for consensus reads using Picard SamToFastq (INTERLEAVE = true, INCLUDE_NON_PF_READS = true). (10). Map consensus reads to reference files of interest using BWA mem -p -t 8 and SAMtools view. (11). Sort unmapped consensus BAM in query name using Picard SortSam. (12). Merge UMI info from unmapped consensus BAM to mapped consensus BAM using Picard MergeBamAlignment (same options as described in step 6).

The Integrative Genomics Viewer (IGV) 2.3.1 [15, 16] [Broad Institute, Cambridge, MA, USA] was used for viewing alignments using bam, bam.bai, fasta, and fasta.fai files as input. SAMtools view -c -F260 was used to estimate the percentage spiked in copyDNA using the FASTA-mapped BAM files produced after pre-alignment to the human reference genome. The number of unique indexes in an index file was counted using a custom script.

## Results

### Dilution series

Plasmids of the potential doping genes *EPO*, *GH1*, *GH2*, *IGF1*, and *IGF2* were mixed with high-molecular gDNA from a pool of donors in a percentage ranging from 0.01 to 1 for this proof-of-principle study. Every sample also contained 1% plasmid *EGFP* for quantification purposes (Table 1). The total number of paired indexed reads passed filter was 18.26 million (Table 1). The median percentage unique unmappable reads to the human_g1K_v37 reference genome was 46% (Table 1). This is a conservative value because the reads can map to different transcripts or different locations, making a duplicated UMI specific for both transcripts or locations.

**Table 1:** Results of sequencing of dilution series

Sample	% of plasmids containing doping genes copyDNA^a^	%Indexed reads passed filter	Number of paired reads (million)	Number of unmappable reads after pre-alignment	%Unique unmappable reads after pre-alignment
1	1% *EPO*	16.5	3.33	248,732	29
2	0.1% *EPO*	14.9	3.01	167,146	44
3	0.01% *EPO*	8.4	1.70	151,759	50
4	1% *GH1*	14.5	2.93	177,547	43
5	0.1% *GH1*	10.5	2.12	161,153	46
6	0.01% *GH1*	8.4	1.70	155,171	49
7	0.1% each *EPO*, *GH1*, *GH2*, *IGF1*, *IGF2*	9.6	1.94	160,385	46
8	0.01% each *EPO*, *GH1*, *GH2*, *IGF1*, *IGF2*	7.6	1.53	222,133	45
Total indexed		90.4	18.26		
Nonindexed reads passed filter		9.6	1.94		

^a^Plasmids of doping genes were mixed with high-molecular gDNA from a pool of donors in percentages ranging from 1–0.01%. One percent EGFP plasmid was added to each sample for quantification purposes

### Analysis strategy 1: alignment of unique unmappable reads to gene-specific reference transcripts

Reads that could not be aligned to the human reference genome because the intron sequences were missing were aligned to fasta files of transcripts of the doping genes, and 0.01% of both *EPO* plasmid and *GH1* plasmid were detected for all exon–exon junctions (Supplementary information 3, tables a and b). There were no false positive results, indicating 100% specificity (Supplementary information 3, tables a and b). Reads were distributed across the exon–exon junctions (Supplementary information 3, tables a and b). Probe performance was not influenced by the presence of other plasmids, as tested by mixing 0.1 and 0.01% *EPO*, *GH1*, *GH2*, *IGF1*, and *IGF2* plasmids (Supplementary information 3, tables a and b). *GH2*, *IGF1*, and *IGF2* were also detectable in the mixing experiments, but were not tested separately (Supplementary information 3, tables a and b).

### Analysis strategy 2: Alignment of all unique reads to gene-specific reference transcripts

In our second analysis, we aligned all unique sequence reads directly to the reference transcripts. Using this analysis, it was possible to detect *EPO* and *GH1* plasmids in percentages far below 0.01% at all exon–exon junctions (Supplementary information 3, tables c and d). The *GH2*, *IGF1,* and *IGF2* plasmids are probably also detectable far below 0.01%, but were only tested in a mixture of multiple plasmids (Supplementary information 3, tables e and f).

### Comparison of analysis strategies 1 and 2

**Fig. 2:**
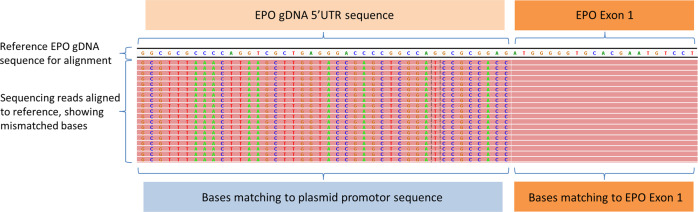
Detection of plasmid sequences by visualization of reads that cannot be aligned to the EPO reference gene in the IGV viewer. Sequencing reads that contain both *EPO* copyDNA and plasmid sequence are shown as partially mismatched reads by visualization of the alignment to *EPO* gDNA in the IGV browser. This allows distinction of *EPO* gDNA and *EPO* copyDNA

**Fig. 3:**
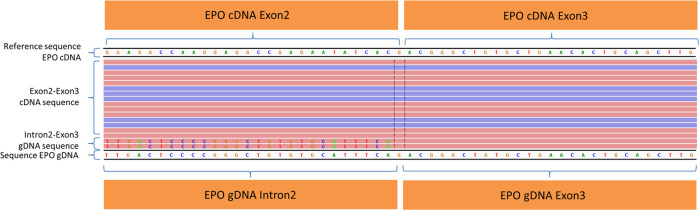
Identification of sequence reads derived from *EPO* gDNA by alignment to *EPO* copyDNA in the IGV viewer. Several reads aligned to the *EPO* copyDNA (cDNA) reference sequence consist of intron–exon or exon–intron sequences derived from *EPO* gDNA as shown in the IGV browser. The mismatches depicted in this figure correspond to the intron 2 sequence of *EPO* gDNA. This allows distinction of *EPO* gDNA and *EPO* cDNA. Forward sequence reads are pink and reverse reads are purple colored

The assigned number of paired reads was divided by the total number of exon–exon junction calls in each sample (Tables 2 and 3). A higher value indicates lower gene-specific fasta file alignment efficiency or a lower concentration of plasmid. Using these values, we compared the different alignment methods. The sensitivity of analysis strategy 2 is much higher than that of analysis strategy 1. Using analysis strategy 1 we detected at least 5 reads per junction (Supplementary information 3, table a) in a 0.01% EPO copyDNA dilution. This is in accordance with a calculated sensitivity of at least 0.002%. Using analysis strategy 2 we counted at least 1000 reads per junction (Supplementary information 3, table c). This is in accordance with a calculated sensitivity of at least 0.0001%. As we added 1,296,800 plasmid copies per 1000 ng in the 0.01% dilution, the expected maximum sensitivity is therefore 1296 copies using analysis strategy 2. However, the specificity of strategy 2 is lower, as shown by alignment of reads with intron–exon junctions originating from gDNA to the reference transcripts (Supplementary information 3, tables c, d and f). However, gDNA and plasmid copyDNA sequences can be distinguished by visualization of the aligned reads in, e.g., the IGV browser, where specific intron regions are easily recognized as mismatched reads (Fig. 2). We manually checked the read alignment of the *EPO* mixtures and observed the absence of the untranslated region in the captured plasmid sequences, indicating that the probes are able to bind sequences that are only partly complementary with sufficient affinity (Fig. 3).

**Table 2:** *EPO* copyDNA detection using sequence reads not mapping to the human reference genome

% Plasmid with doping genes cDNA	Number of EJ *EPO* total	Number of EJ *GH1* total	Number of paired reads (million)	Fraction^a^ EJ *EPO*	Fraction^a^ EJ *GH1*
1% *EPO*	15,352	0	3.33	217	
0.1% *EPO*	2644	0	3.01	1138	
0.01% *EPO*	143	0	1.70	11,888	
1% *GH1*	0	12,302	2.93		238
0.1% *GH1*	0	970	2.12		2185
0.01% *GH1*	0	57	1.70		29,825

*EJ* exon–exon junctions

^a^Fraction is calculated by dividing number of paired reads by EJ doping gene total

**Table 3:** *EPO* copyDNA detection using all unique sequence reads

% Plasmid with doping genes cDNA	EJ *EPO* total	EJ *GH1* total	Paired reads (million)	Fraction^a^ EJ *EPO*	Fraction^a^ EJ *GH1*
1% *EPO*	916,544	0	3.33	4	
0.1% *EPO*	112,251	0	3.01	27	
0.01% *EPO*	5741	0	1.70	296	
1% *GH1*	0	909,954	2.93		3
0.1% *GH1*	0	70,929	2.12		30
0.01% *GH1*	0	5312	1.70		320

*EJ* exon–exon junctions

^a^Fraction is calculated by dividing number of paired reads by EJ doping gene total

### Quantification of gene doping plasmid using EGFP plasmid

We spiked a standard of 1% *EGFP* plasmid in every sample to enable quantification of the percentage of gene doping plasmid. We used analysis strategy 1 to test the performance of this quantification method because a higher specificity will increase the accuracy of quantification. *EGFP* read counts were constantly higher than *EPO* read counts as expected due to the design of the probes: (1). Probes for *EGFP* were tiled to reach the maximum coverage and because of this the highest accuracy while *EPO* probes were only directed to the exon–exon junctions, (2). CopyDNA *EPO* is in competition with gDNA and *EGFP* has no endogenous competition. We found stable numbers of *EGFP* sequences across samples, enabling quantification of *EPO* plasmid levels. The ratios detected, once converted to percentages, are close to the percentage of plasmid added to the samples (Table 4). This method appeared to work for all the doping genes tested, but *EPO* is the best example to show because the quantification of *EPO* is not influenced by the presence of homologous genes.

**Table 4:** Quantification of gene doping copyDNA

% Plasmid containing *EPO* copyDNA	Reads *EPO* copyDNA	Reads *EGFP* copyDNA^a^	Ratio^b^ *EPO*-*EGFP*
1	15,238	2,323,581	0.0066
0.1	2638	2,370,910	0.0011
0.01	137	1,360,263	0.00010
0	0	2,047,770	0
0	0	1,758,981	0
0	0	1,415,380	0
0.1^c^	2085	1,528,579	0.0014
0.01^c^	199	1,275,941	0.00016

^a^the percentage *EGFP* plasmid is added in a standard concentration of 1% in all samples

^b^Ratio is calculated by dividing the number of EPO reads by the number of EGFP reads

^c^Mixed with other plasmids in one sample

## Discussion

Gene doping-derived proteins produced by the body of the athlete would, in most cases, be indistinguishable from endogenous proteins, and detection of gene doping should therefore take place at the DNA level [5–7, 10]. Transgenic gene constructs are distinguishable from gDNA through the existence of exon–exon junctions because gene-therapy vectors will use copyDNA due to its smaller size compared with gDNA [10]. We have therefore developed and validated an NGS-based gene doping detection panel that is applicable to plasmid- and virus-derived copyDNA sequences and intact constructs (transgenic gene-constructs) in a huge excess of gDNA. By starting with a DNA isolation and using adapters that are only able to ligate to double stranded fragments, we eliminate RNA molecules, which also contain exon–exon junctions that might interfere with the detection resulting in less sensitivity.

Our NGS panel allows simultaneous detection of multiple potential doping genes in one sample using a single platform. This panel targets all exon–exon junctions of all transcripts of the genes *EPO*, *IGF1*, *IGF2 GH1*, and *GH2*. However, it can also be easily expanded to detect other genes by supplementing with additional capturing probes. Doing so will not affect detection of the genes already in the panel and will still enable detection with high specificity in the presence of transgenic gene constructs of different genes in one sample (as we have already shown for the current panel). During sample preparation, sample-specific barcodes and molecular indexes are added to each individual DNA molecule to allow for multiplexing of samples and removal of duplicated reads and PCR-induced sequence artifacts, respectively, which increases specificity. Finally, we were able to quantify gene doping copyDNA levels by spiking fixed amounts of *EGFP* plasmid into each sample, resulting in stable amounts of read counts across samples.

LAMP and PCR-based gene doping detection methods (like real-time PCR, nested PCR, droplet digital PCR and internal threshold PCR) target exon–exon junctions of transgenic gene constructs similarly to our NGS-based method [3, 5–13]. However, these methods need at least one wild-type copyDNA junction for detection of gene doping [6, 8, 9]. Dependence on a limited number of junctions with a fixed sequence gives little flexibility in the design of the short length PCR primers and probes [8]. To avoid detection, gene doping suppliers could reduce the number of targetable junctions by tampering with the copyDNA sequences. Exon–exon junctions could, for example, be manipulated by introducing silent mutations with no consequences at protein level that interfere with PCR primer and probe annealing, and thus detection [10]. Our NGS-based detection method uses much longer capturing probes that are able to bind junctions that are only partly complementary with sufficient affinity for capturing. This makes our method far less sensitive to tampering through alteration of the copyDNA sequence. We did not test the capturing efficiency of the panel in a situation where many silent mutations are introduced. Further experiments are needed to show if the sensitivity to detect such alternative sequences is equal to that of the untampered sequence. However, probes targeting alternative sequences can be added to the panel without affecting the performance of the probes already in the panel. Our method allows users to check the actual copyDNA sequences in the sequencing data to readily detect manipulation of sequences, promotor regions and plasmid-derived sequences. For instance, we were able to detect nontranslated 5′ plasmid-derived sequences. Having knowledge of the actual copyDNA sequence could then give authorities the opportunity to develop conventional PCR-based technologies for independent secondary tests for confirmation of positive doping detection.

It is still unknown how many copies of gene doping copyDNA will be present in an athlete’s circulation at a given time after administration, so we do not know how sensitive gene doping detection needs to be. It has been described that a variable shedding of the vector and the biological distribution depends on a lot of factors like delivery route, the type of vector and the sample origin [10, 17, 18]. Because of these uncertainties the sensitivity needs to be as high as possible. Previously published studies on gene doping detection have focused on the maximally achievable sensitivity of their methods. PCR-based methods for gene doping detection report a sensitivity of about 4–14 copies of gene doping copyDNA in 1000 ng whole-blood-isolated human gDNA [3]. Our NGS-based method currently reaches a sensitivity of 1296 copies in 1000 ng gDNA using the strategy of direct alignment of all unique reads to gene-specific reference transcripts, which is ~100-times lower than PCR-based methods. The method can further be optimized to increase the sensitivity by increasing the percentage of copyDNA fragments in the captured library. One way to do this is to isolate DNA (copyDNA and gDNA) from blood plasma instead of whole blood, similar to what we have done for noninvasive prenatal testing [19]. The percentage of gDNA compared with copyDNA in plasma is far lower than that in whole blood because of the removal of white blood cells. Alternatively, we could increase the capturing efficiency by specific blocking of gDNA sequences during the capturing process with nonbiotinylated probes. These future adjustments will improve sensitivity and lower the costs since fewer reads are needed to detect each gene doping copyDNA transcript.

Another future possibility for gene editing in sports is the use of cluster regularly interspaced short palindromic repeats (CRISPR)-Cas (CRISPR-associated) [20]. CRISPR-Cas can for example be used to disrupt regulatory genes, such as Myostatin, a negative regulator of muscle growth [21]. A major risk of CRISPR-Cas are the off-target effects in human cells [20] and therefore we do not expect it to be used in the near future for this purpose. For this type of gene doping to be detected, modification of our method will be required by changing the input material to RNA (converting to complementary DNA) and adding probes targeting genes of interest and house-keeping genes. In this way our method can detect induced alterations in gene expression.

We cannot completely rule out that our NGS-based test will capture gDNA and that these fragments will be sequenced, which could raise concerns about disclosing genetic information and privacy [22]. However, we are sure that we can maximally limit these concerns. Unsolicited findings that might raise ethical dilemmas are excluded by capturing only the genomic sequences of the doping genes and by only mapping captured reads to specific gene doping reference genomes. Genomic sequences of doping genes were the only unsolicited findings after alignment to the specific gene doping reference genomes. Genomic fingerprinting of intragenic genomic areas can be used to prove that a result belongs to a specific person and to exclude contamination. This method will be further developed in a real-life situation.

To summarize, our method outperforms existing PCR-based methods in many aspects and can be further developed into a routine method for detection of gene doping of multiple genes that can be used in all sports. The method needs to be implemented in routine doping laboratories with the right infrastructure.

## Supplementary information

Supplementary Information
