# Supplementary material for: A next-generation sequencing method for gene doping detection that distinguishes low levels of plasmid DNA against a background of genomic DNA
Source: Gene Ther. 2019 Jul 11;26(7-8):338–46. doi: 10.1038/s41434-019-0091-6 (PMC6760532; doi:10.1038/s41434-019-0091-6)
Supplement: Supplementary file 1 — Supplementary Information [file 41434_2019_91_MOESM1_ESM.docx]

**Supplementary information 1: Plasmids with junctions**

The plasmids pcDNA3.1+/C-(K)DYK-EPO, pcDNA3.1+/C-(K)DYK-GH1, pcDNA3.1+/C-(K)DYK-GH2, pcDNA3.1+/C (K)DYK-IGF1 and pcDNA3.1+/C-(K)DYK-IGF2 [Genescript, Piscataway Township, NJ, USA] were used for validating the method. The exon-exon junctions in the plasmids are underlined.

*EPO*

ATGGGGGTGCACGAATGTCCTGCCTGGCTGTGGCTTCTCCTGTCCCTGCTGTCGCTCCCTCTGGGCCTCCCAGTCCTGGGCGCCCCACCACGCCTCATCTGTGACAGCCGAGTCCTGGAGAGGTACCTCTTGGAGGCCAAGGAGGCCGAGAATATCACGACGGGCTGTGCTGAACACTGCAGCTTGAATGAGAATATCACTGTCCCAGACACCAAAGTTAATTTCTATGCCTGGAAGAGGATGGAGGTCGGGCAGCAGGCCGTAGAAGTCTGGCAGGGCCTGGCCCTGCTGTCGGAAGCTGTCCTGCGGGGCCAGGCCCTGTTGGTCAACTCTTCCCAGCCGTGGGAGCCCCTGCAGCTGCATGTGGATAAAGCCGTCAGTGGCCTTCGCAGCCTCACCACTCTGCTTCGGGCTCTGGGAGCCCAGAAGGAAGCCATCTCCCCTCCAGATGCGGCCTCAGCTGCTCCACTCCGAACAATCACTGCTGACACTTTCCGCAAACTCTTCCGAGTCTACTCCAATTTCCTCCGGGGAAAGCTGAAGCTGTACACAGGGGAGGCCTGCAGGACAGGGGACAGA

*GH1*

ATGGCTACAGGCTCCCGGACGTCCCTGCTCCTGGCTTTTGGCCTGCTCTGCCTGCCCTGGCTTCAAGAGGGCAGTGCCTTCCCAACCATTCCCTTATCCAGGCTTTTTGACAACGCTATGCTCCGCGCCCATCGTCTGCACCAGCTGGCCTTTGACACCTACCAGGAGTTTGAAGAAGCCTATATCCCAAAGGAACAGAAGTATTCATTCCTGCAGAACCCCCAGACCTCCCTCTGTTTCTCAGAGTCTATTCCGACACCCTCCAACAGGGAGGAAACACAACAGAAATCCAACCTAGAGCTGCTCCGCATCTCCCTGCTGCTCATCCAGTCGTGGCTGGAGCCCGTGCAGTTCCTCAGGAGTGTCTTCGCCAACAGCCTGGTGTACGGCGCCTCTGACAGCAACGTCTATGACCTCCTAAAGGACCTAGAGGAAGGCATCCAAACGCTGATGGGGAGGCTGGAAGATGGCAGCCCCCGGACTGGGCAGATCTTCAAGCAGACCTACAGCAAGTTCGACACAAACTCACACAACGATGACGCACTACTCAAGAACTACGGGCTGCTCTACTGCTTCAGGAAGGACATGGACAAGGTCGAGACATTCCTGCGCATCGTGCAGTGCCGCTCTGTGGAGGGCAGCTGTGGCTTCTAG

*GH2*

ATGGCTGCAGGCTCCCGGACGTCCCTGCTCCTGGCTTTTGGCCTGCTCTGCCTGTCCTGGCTTCAAGAGGGCAGTGCCTTCCCAACCATTCCCTTATCCAGGCTTTTTGACAACGCTATGCTCCGCGCCCGTCGCCTGTACCAGCTGGCATATGACACCTATCAGGAGTTTGAAGAAGCCTATATCCTGAAGGAGCAGAAGTATTCATTCCTGCAGAACCCCCAGACCTCCCTCTGCTTCTCAGAGTCTATTCCAACACCTTCCAACAGGGTGAAAACGCAGCAGAAATCTAACCTAGAGCTGCTCCGCATCTCCCTGCTGCTCATCCAGTCATGGCTGGAGCCCGTGCAGCTCCTCAGGAGCGTCTTCGCCAACAGCCTGGTGTATGGCGCCTCGGACAGCAACGTCTATCGCCACCTGAAGGACCTAGAGGAAGGCATCCAAACGCTGATGTGGAGGCTGGAAGATGGCAGCCCCCGGACTGGGCAGATCTTCAATCAGTCCTACAGCAAGTTTGACACAAAATCGCACAACGATGACGCACTGCTCAAGAACTACGGGCTGCTCTACTGCTTCAGGAAGGACATGGACAAGGTCGAGACATTCCTGCGCATCGTGCAGTGCCGCTCTGTGGAGGGCAGCTGTGGCTTCTAG

*IGF1*

ATGGGAAAAATCAGCAGTCTTCCAACCCAATTATTTAAGTGCTGCTTTTGTGATTTCTTGAAGGTGAAGATGCACACCATGTCCTCCTCGCATCTCTTCTACCTGGCGCTGTGCCTGCTCACCTTCACCAGCTCTGCCACGGCTGGACCGGAGACGCTCTGCGGGGCTGAGCTGGTGGATGCTCTTCAGTTCGTGTGTGGAGACAGGGGCTTTTATTTCAACAAGCCCACAGGGTATGGCTCCAGCAGTCGGAGGGCGCCTCAGACAGGCATCGTGGATGAGTGCTGCTTCCGGAGCTGTGATCTAAGGAGGCTGGAGATGTATTGCGCACCCCTCAAGCCTGCCAAGTCAGCTCGCTCTGTCCGTGCCCAGCGCCACACCGACATGCCCAAGACCCAGAAGTATCAGCCCCCATCTACCAACAAGAACACGAAGTCTCAGAGAAGGAAAGGAAGTACATTTGAAGAACGCAAGTAG

*IGF2*

ATGGGAATCCCAATGGGGAAGTCGATGCTGGTGCTTCTCACCTTCTTGGCCTTCGCCTCGTGCTGCATTGCTGCTTACCGCCCCAGTGAGACCCTGTGCGGCGGGGAGCTGGTGGACACCCTCCAGTTCGTCTGTGGGGACCGCGGCTTCTACTTCAGCAGGCCCGCAAGCCGTGTGAGCCGTCGCAGCCGTGGCATCGTTGAGGAGTGCTGTTTCCGCAGCTGTGACCTGGCCCTCCTGGAGACGTACTGTGCTACCCCCGCCAAGTCCGAGAGGGACGTGTCGACCCCTCCGACCGTGCTTCCGGACAACTTCCCCAGATACCCCGTGGGCAAGTTCTTCCAATATGACACCTGGAAGCAGTCCACCCAGCGCCTGCGCAGGGGCCTGCCTGCCCTCCTGCGTGCCCGCCGGGGTCACGTGCTCGCCAAGGAGCTCGAGGCGTTCAGGGAGGCCAAACGTCACCGTCCCCTGATTGCTCTACCCACCCAAGACCCCGCCCACGGGGGCGCCCCCCCAGAGATGGCCAGCAATCGGAAGTGA

**Supplementary information 2: Probe sequences**

Biotin-labeled probes designed against all protein coding exon-exon junctions. The plasmid *EGFP* sequence was fully tiled with probes. Sequences and corresponding information are collected in this attachment. Corresponding information contains (successively): gene name, probe number, amount of bp exon x, amount of bp exon y.

>*EPO*_1 _60_60

CACCCGGCGCGCCCCAGGTCGCTGAGGGACCCCGGCCAGGCGCGGAGATGGGGGTGCACGAATGTCCTGCCTGGCTGTGGCTTCTCCTGTCCCTGCTGTCGCTCCCTCTGGGCCTCCCAG

>*EPO*_2_60_60

AACTTTGGTGTCTGGGACAGTGATATTCTCATTCAAGCTGCAGTGTTCAGCACAGCCCGTCGTGATATTCTCGGCCTCCTTGGCCTCCAAGAGGTACCTCTCCAGGACTCGGCTGTCACA

>*EPO*_3_40_80

AGACACCAAAGTTAATTTCTATGCCTGGAAGAGGATGGAGGTCGGGCAGCAGGCCGTAGAAGTCTGGCAGGGCCTGGCCCTGCTGTCGGAAGCTGTCCTGCGGGGCCAGGCCCTGTTGGT

>*EPO*_4_60_60

AGCAGTGATTGTTCGGAGTGGAGCAGCTGAGGCCGCATCTGGAGGGGAGATGGCTTCCTTCTGGGCTCCCAGAGCCCGAAGCAGAGTGGTGAGGCTGCGAAGGCCACTGACGGCTTTATC

>*IGF2*_1_60_60

TACTGTGCTACCCCCGCCAAGTCCGAGAGGGACGTGTCGACCCCTCCGACCGTGCTTCCGGACAACTTCCCCAGATACCCCGTGGGCAAGTTCTTCCAATATGACACCTGGAAGCAGTCC

>*IGF2*_2_60_60

GCGGCGGGGAGCTGGTGGACACCCTCCAGTTCGTCTGTGGGGACCGCGGCTTCTACTTCAGCAGGCCCGCAAGCCGTGTGAGCCGTCGCAGCCGTGGCATCGTTGAGGAGTGCTGTTTCC

>*IGF2*_3_60_60

GCGGCGGGGAGCTGGTGGACACCCTCCAGTTCGTCTGTGGGGACCGCGGCTTCTACTTCAGACTTCCAGGCAGGCCCGCAAGCCGTGTGAGCCGTCGCAGCCGTGGCATCGTTGAGGAGT

>*IGF2*_4_60_60

TCCTCCTCCTCCTCTTCCTCCTCCTCCTCCTGCCCCAGCGAGCCTTCTGCTGAGCTGTAGACACCAATGGGAATCCCAATGGGGAAGTCGATGCTGGTGCTTCTCACCTTCTTGGCCTTC

>*IGF2*_5_60_60

AGCTTCCTCCTCCTCCTCTTCCTCCTCCTCCTCCTGCCCCAGCGAGCCTTCTGCTGAGCTACACCAATGGGAATCCCAATGGGGAAGTCGATGCTGGTGCTTCTCACCTTCTTGGCCTTC

>*IGF2*_6_60_60

CCCGCTGTTCGGTTTGCGACACGCAGCAGGGAGGTGGGCGGCAGCGTCGCCGGCTTCCAGACACCAATGGGAATCCCAATGGGGAAGTCGATGCTGGTGCTTCTCACCTTCTTGGCCTTC

>*IGF2*_7_60_60

TTTTGGGTGGGCCGCAAAGGCGAGCTACTTAGACGCACCCCGGTGAGCTCGGCCATGCAGACACCAATGGGAATCCCAATGGGGAAGTCGATGCTGGTGCTTCTCACCTTCTTGGCCTTC

>*IGF2*_8_60_60

GTTCGATCGCTCGCTGCCTGAGCTCCTGGTGCGCCCGCGGACGCAGCCTCCAGCTTCGCGGAGATGGTTTCCCCAGACCCCCAAATTATCGTGGTGGCCCCCGAGACCGAACTCGCGTCT

>*IGF2*_9_60_60

GTTCGATCGCTCGCTGCCTGAGCTCCTGGTGCGCCCGCGGACGCAGCCTCCAGCTTCGCGACACCAATGGGAATCCCAATGGGGAAGTCGATGCTGGTGCTTCTCACCTTCTTGGCCTTC

>*IGF2*_10_60_60

GCTCCCAGAACTGAGGCTGGCAGCCAGCCCCAGCCTCAGCCCCAACTGCGAGGCAGAGAGACACCAATGGGAATCCCAATGGGGAAGTCGATGCTGGTGCTTCTCACCTTCTTGGCCTTC

>*IGF2*_11_60_60

CCCGGTCCTCTTTATCCACTGTCCAGGAGCTGCGGGGACTGCGCAGGGACTAGAGTACAGGGGCCGAAGAGTCACCACCGAGCTTGTGTGGGAGGAGGTGGATTCCAGCCCCCAGCCCCA

>*IGF1*_1_40_49_31

CCGTGCCCAGCGCCACACCGACATGCCCAAGACCCAGAAGTATCAGCCCCCATCTACCAACAAGAACACGAAGTCTCAGAGAAGGAAAGGAAGTACATTTGAAGAACGCAAGTAGAGGGA

>*IGF1*_2_60_60

GCCAAGTCAGCTCGCTCTGTCCGTGCCCAGCGCCACACCGACATGCCCAAGACCCAGAAGGAAGTACATTTGAAGAACGCAAGTAGAGGGAGTGCAGGAAACAAGAACTACAGGATGTAG

>*IGF1*_3_60_60

GCCAAGTCAGCTCGCTCTGTCCGTGCCCAGCGCCACACCGACATGCCCAAGACCCAGAAGTATCAGCCCCCATCTACCAACAAGAACACGAAGTCTCAGAGAAGGAAAGGTTGGCCAAAG

>*IGF1*_4_60_60

CATCCACGATGCCTGTCTGAGGCGCCCTCCGACTGCTGGAGCCATACCCTGTGGGCTTGTTGAAATAAAAGCCCCTGTCTCCACACACGAACTGAAGAGCATCCACCAGCTCAGCCCCGC

>*IGF1*_5_60_60

TAATACCCACCCTGACCTGCTGTAAAAGACCTGGAACAAACAAAAATGATTACACCTACAGTGAAGATGCACACCATGTCCTCCTCGCATCTCTTCTACCTGGCGCTGTGCCTGCTCACC

>*IGF1*_6_60_60

GGAAAAATCAGCAGTCTTCCAACCCAATTATTTAAGTGCTGCTTTTGTGATTTCTTGAAGGTGAAGATGCACACCATGTCCTCCTCGCATCTCTTCTACCTGGCGCTGTGCCTGCTCACC

>*GH2*_1_60_60

AAACTTGCTGTAGGACTGATTGAAGATCTGCCCAGTCCGGGGGCTGCCATCTTCCAGCCTCCACATCAGCGTTTGGATGCCTTCCTCTAGGTCCTTCAGGTGGCGATAGACGTTGCTGTC

>*GH2*_2_60_60

AAACTTGCTGTAGGACTGATTGAAGATCTGCCCAGTCCGGGGGCTGCCATCTTCCAGCCTATCAGCGTTTGGATGCCTTCCTCTAGGTCCTTCAGGTGGCGATAGACGTTGCTGTCCGAG

>*GH2*_3_35_85

CACCTTCCAACAGGGTGAAAACGCAGCAGAAATCTAACCTAGAGCTGCTCCGCATCTCCCTGCTGCTCATCCAGTCATGGCTGGAGCCCGTGCAGCTCCTCAGGAGCGTCTTCGCCAACA

>*GH2*_4_55_65

CTTCTCAGAGTCTATTCCAACACCTTCCAACAGGGTGAAAACGCAGCAGAAATCTAACCTAGAGCTGCTCCGCATCTCCCTGCTGCTCATCCAGTCATGGCTGGAGCCCGTGCAGCTCCT

>*GH2*_5_35_85

ATAGACTCTGAGAAGCAGAGGGAGGTCTGGGGGTTAAACTCCTGATAGGTGTCATATGCCAGCTGGTACAGGCGACGGGCGCGGAGCATAGCGTTGTCAAAAAGCCTGGATAAGGGAATG

>*GH2*_6_60_60

GGAGGTCTGGGGGTTCTGCAGGAATGAATACTTCTGCTCCTTCAGGATATAGGCTTCTTCAAACTCCTGATAGGTGTCATATGCCAGCTGGTACAGGCGACGGGCGCGGAGCATAGCGTT

>*GH2*_7_51_69

CCGAACCACTCAGGGTCCTGTGGACAGCTCACCTAGCGGCAATGGCTGCAGGCTCCCGGACGTCCCTGCTCCTGGCTTTTGGCCTGCTCTGCCTGTCCTGGCTTCAAGAGGGCAGTGCCT

>*GH2*_8_60_60

CCCAACTCCCCGAACCACTCAGGGTCCTGTGGACAGCTCACCTAGCGGCAATGGCTGCAGGCTCCCGGACGTCCCTGCTCCTGGCTTTTGGCCTGCTCTGCCTGTCCTGGCTTCAAGAGG

>*GH1*_1_38_82

TAAAGGACCTAGAGGAAGGCATCCAAACGCTGATGGGGAGGCTGGAAGATGGCAGCCCCCGGACTGGGCAGATCTTCAAGCAGACCTACAGCAAGTTCGACACAAACTCACACAACGATG

>*GH1*_2_60_60

GACAGCAACGTCTATGACCTCCTAAAGGACCTAGAGGAAGGCATCCAAACGCTGATGGGGAGGCTGGAAGATGGCAGCCCCCGGACTGGGCAGATCTTCAAGCAGACCTACAGCAAGTTC

>*GH1*_3_11_109

AACAGAAATCCAACCTAGAGCTGCTCCGCATCTCCCTGCTGCTCATCCAGTCGTGGCTGGAGCCCGTGCAGTTCCTCAGGAGTGTCTTCGCCAACAGCCTGGTGTACGGCGCCTCTGACA

>*GH1*_4_41_79

TTCCGACACCCTCCAACAGGGAGGAAACACAACAGAAATCCAACCTAGAGCTGCTCCGCATCTCCCTGCTGCTCATCCAGTCGTGGCTGGAGCCCGTGCAGTTCCTCAGGAGTGTCTTCG

>*GH1*_5_41_79

ATCGTCTGCACCAGCTGGCCTTTGACACCTACCAGGAGTTTAGGCTGGAAGATGGCAGCCCCCGGACTGGGCAGATCTTCAAGCAGACCTACAGCAAGTTCGACACAAACTCACACAACG

>*GH1*_6_60_60

AACGCTATGCTCCGCGCCCATCGTCTGCACCAGCTGGCCTTTGACACCTACCAGGAGTTTGAAGAAGCCTATATCCCAAAGGAACAGAAGTATTCATTCCTGCAGAACCCCCAGACCTCC

>*GH1*_7_60_60

AACGCTATGCTCCGCGCCCATCGTCTGCACCAGCTGGCCTTTGACACCTACCAGGAGTTTAACCCCCAGACCTCCCTCTGTTTCTCAGAGTCTATTCCGACACCCTCCAACAGGGAGGAA

>*GH1*_8_60_60

AACGCTATGCTCCGCGCCCATCGTCTGCACCAGCTGGCCTTTGACACCTACCAGGAGTTTAACCTAGAGCTGCTCCGCATCTCCCTGCTGCTCATCCAGTCGTGGCTGGAGCCCGTGCAG

>*GH1*_9_110_10

CATAGCGTTGTCAAAAAGCCTGGATAAGGGAATGGTTGGGAAGGCACTGCCCTCTTGAAGCCAGGGCAGGCAGAGCAGGCCAAAAGCCAGGAGCAGGGACGTCCGGGAGCCTGTAGCCAT

>*GH1*_10_67_53

GCACTGCCCTCTTGAAGCCAGGGCAGGCAGAGCAGGCCAAAAGCCAGGAGCAGGGACGTCCGGGAGCCTGTAGCCATTGCAGCTAGGTGAGCTGTCCACAGGACCCTGAGTGGTTCGGGG

>Plasmid*EGFP*

CTTTTGCAAAGATCGATCAAGAGACAGGATGAGGATCGTTTCGCATGATTGAACAAGATGGATTGCACGCAGGTTCTCCGGCCGCTTGGGTGGAGAGGCTATTCGGCTATGACTGGGCACAACAGACAATCGGCTGCTCTGATGCCGCCGTGTTCCGGCTGTCAGCGCAGGGGCGCCCGGTTCTTTTTGTCAAGACCGACCTGTCCGGTGCCCTGAATGAACTGCAAGACGAGGCAGCGCGGCTATCGTGGCTGGCCACGACGGGCGTTCCTTGCGCAGCTGTGCTCGACGTTGTCACTGAAGCGGGAAGGGACTGGCTGCTATTGGGCGAAGTGCCGGGGCAGGATCTCCTGTCATCTCACCTTGCTCCTGCCGAGAAAGTATCCATCATGGCTGATGCAATGCGGCGGCTGCATACGCTTGATCCGGCTACCTGCCCATTCGACCACCAAGCGAAACATCGCATCGAGCGAGCACGTACTCGGATGGAAGCCGGTCTTGTCGATCAGGATGATCTGGACGAAGAGCATCAGGGGCTCGCGCCAGCCGAACTGTTCGCCAGGCTCAAGGCGAGCATGCCCGACGGCGAGGATCTCGTCGTGACCCATGGCGATGCCTGCTTGCCGAATATCATGGTGGAAAATGGCCGCTTTTCTGGATTCATCGACTGTGGCCGGCTGGGTGTGGCGGACCGCTATCAGGACATAGCGTTGGCTACCCGTGATATTGCTGAAGAGCTTGGCGGCGAATGGGCTGACCGCTTCCTCGTGCTTTACGGTATCGCCGCTCCCGATTCGCAGCGCATCGCCTTCTATCGCCTTCTTGACGAGTTCTTCTGAGCGGGACTCTGGGGTTCGAAATGACCGACCAAGCGACGCCCAACCTGCCATCACGAGATTTCGATTCCACCGCCGCCTTCTATGAAAGGTTGGGCTTCGGAATCGTTTTCCGGGACGCCGGCTGGATGATCCTCCAGCGCGGGGATCTCATGCTGGAGTTCTTCGCCCAC

**Supplementary information 3: Tables showing sensitivity and specificity of the gene doping detection assay**

**Table a:** *EPO* copyDNA detection using sequence reads not mapping to the human reference genome

| **Sample** | **% Plasmid containing doping genes copyDNA** | **EJ1^a^** | **EJ2** | **EJ3** | **EJ4** |
| --- | --- | --- | --- | --- | --- |
| **1** | 1% *EPO* | 8323 | 4875 | 1188 | 966 |
| **2** | 0.1% *EPO* | 1386 | 870 | 219 | 169 |
| **3** | 0.01% *EPO* | 81 | 46 | 11 | 5 |
| **4** | 1% *GH1* | 0 | 0 | 0 | 0 |
| **5** | 0.1% *GH1* | 0 | 0 | 0 | 0 |
| **6** | 0.01% *GH1* | 0 | 0 | 0 | 0 |
| **7^b^** | 0.1% each of *EPO*, *GH1*, *GH2*, *IGF1*, *IGF2* | 1031 | 734 | 228 | 138 |
| **8^b^** | 0.01% each of *EPO*, *GH1*, *GH2*, *IGF1*, *IGF2* | 94 | 81 | 22 | 11 |

^a^Plasmid does not contain UTR region, only 73 bp of the probe sequence in common with the plasmid sequence

^b^Mixed with *GH1*, *GH2*, *IGF1* and *IGF2* plasmid in the same percentage.

EJ1 = exon-exon junction 1 = *EPO*_ENST00000252723_ENSE00001130431-ENSE00001144077

EJ2 = exon-exon junction 2 = *EPO*_ENST00000252723_ENSE00001144077-ENSE00001130423

EJ3 = exon-exon junction 3 = *EPO*_ENST00000252723_ENSE00001130423-ENSE00001130416

EJ4 = exon-exon junction 4 = *EPO*_ENST00000252723_ENSE00001130416-ENSE00000894545

**Table b:** *GH1* copyDNA detection using sequence reads not mapping to the human reference genome

| **Sample** | **% Plasmid containing doping genes copyDNA** | **EJ1** | **EJ2** | **EJ3^a^** | **EJ4** |
| --- | --- | --- | --- | --- | --- |
| **4** | 1% GH1 | 6455 | 3638 | 372 | 1837 |
| **5** | 0.1% GH1 | 494 | 290 | 38 | 148 |
| **6** | 0.01% GH1 | 26 | 20 | 2 | 9 |
| **1** | 0% GH1 | 0 | 0 | 0 | 0 |
| **2** | 0% GH1 | 0 | 0 | 0 | 0 |
| **3** | 0% GH1 | 0 | 0 | 0 | 0 |
| **7^b^** | 0.1% each of EPO, GH1, GH2, IGF1, IGF2 | 454 | 267 | 37 | 182 |
| **8^b^** | 0.01% each of EPO, GH1, GH2, IGF1, IGF2 | 9 | 9 | 0 | 0 |

^a^Plasmid sequence left of the junction 41 bp in common with probe sequence

^b^Mixed with *GH1*, *GH2*, *IGF1* and *IGF2* plasmid in the same percentage.

EJ1 = exon-exon junction 1 = *GH1*_ENST00000323322_ENSE00002549577-ENSE00002430246

EJ2 = exon-exon junction 2 = *GH1*_ENST00000323322_ENSE00002430246-ENSE00002498148

EJ3 = exon-exon junction 3 = *GH1*_ENST00000323322_ENSE00002498148-ENSE00003464202

EJ4 = exon-exon junction 4 = *GH1*_ENST00000323322_ENSE00003464202-ENSE00002220865

**Table c:** *EPO* copyDNA detection using all unique sequence reads

| **Sample** | | **EJ1^a^** | | | | **EJ2** | | | | **EJ3** | | | | **EJ4** | | | |
| --- | --- | --- | --- | --- | --- | --- | --- | --- | --- | --- | --- | --- | --- | --- | --- | --- | --- |
| **%EPO** | **Total** | | **I-E/E-I** | | **Total** | | **I-E/E-I** | | **Total** | | **I-E/E-I** | | **Total** | | **I-E/E-I** | |  |
| **1 (1)** | | 160887 | | 2413 | | 279524 | | 4192 | | 288323 | | 4324 | | 201765 | | 3026 | |
| **2 (0.1)** | | 20993 | | 1154 | | 34877 | | 1918 | | 36570 | | 2011 | | 26342 | | 1448 | |
| **3 (0.01)** | | 2059 | | 803 | | 2363 | | 916 | | 2349 | | 916 | | 2631 | | 1026 | |
| **4 (0)** | | 916 | | 916 | | 839 | | 839 | | 775 | | 775 | | 1526 | | 1526 | |
| **5 (0)** | | 1059 | | 1059 | | 815 | | 815 | | 823 | | 823 | | 1791 | | 1791 | |
| **6 (0)** | | 905 | | 905 | | 807 | | 807 | | 796 | | 796 | | 1594 | | 1594 | |

^a^Plasmid does not contain UTR region, only 73 bp of the probe sequence in common with the plasmid sequence

EJ1 = exon-exon junction 1 = *EPO*_ENST00000252723_ENSE00001130431-ENSE00001144077

EJ2 = exon-exon junction 2 = *EPO*_ENST00000252723_ENSE00001144077-ENSE00001130423

EJ3 = exon-exon junction 3 = *EPO*_ENST00000252723_ENSE00001130423-ENSE00001130416

EJ4 = exon-exon junction 4 = *EPO*_ENST00000252723_ENSE00001130416-ENSE00000894545

Total = total reads aligned

I-E/E-I = genomic DNA specific intron-exon or exon-intron sequences

**Table d:** *GH1* copyDNA detection using all unique sequence reads

| **Sample** | | **EJ1** | | | | **EJ2** | | | | **EJ3^a^** | | | | **EJ4** | | | |
| --- | --- | --- | --- | --- | --- | --- | --- | --- | --- | --- | --- | --- | --- | --- | --- | --- | --- |
| **%GH1** | **Total** | | **I-E/E-I** | | **Total** | | **I-E/E-I** | | **Total** | | **I-E/E-I** | | **Total** | | **I-E/E-I** | |  |
| **4 (1)** | | 156450 | | 3442 | | 243406 | | 5355 | | 256012 | | 5632 | | 274555 | | 6040 | |
| **5 (0.1)** | | 14362 | | 1795 | | 20507 | | 2563 | | 21381 | | 2673 | | 24811 | | 3101 | |
| **6 (0.01)** | | 3619 | | 2208 | | 3082 | | 1880 | | 2551 | | 1556 | | 4368 | | 2664 | |
| **1 (0)** | | 4029 | | 4029 | | 2223 | | 2223 | | 1235 | | 1235 | | 3518 | | 3518 | |
| **2 (0)** | | 4879 | | 4879 | | 3077 | | 3077 | | 1700 | | 1700 | | 4725 | | 4725 | |
| **3 (0)** | | 2938 | | 2938 | | 1811 | | 1811 | | 1050 | | 1050 | | 2790 | | 2790 | |

^a^Plasmid sequence left of the junction 41 bp in common with probe sequence

EJ1 = exon-exon junction 1 = *GH1*_ENST00000323322_ENSE00002549577-ENSE00002430246

EJ2 = exon-exon junction 2 = *GH1*_ENST00000323322_ENSE00002430246-ENSE00002498148

EJ3 = exon-exon junction 3 = *GH1*_ENST00000323322_ENSE00002498148-ENSE00003464202

EJ4 = exon-exon junction 4 = *GH1*_ENST00000323322_ENSE00003464202-ENSE00002220865

Total = total reads aligned

I-E/E-I = genomic DNA specific intron-exon or exon-intron sequences

**Table e:** *GH2*, *IGF1*, *IGF2* copyDNA detection using sequence reads not mapping to the human reference genome

| **Gene** | **% Plasmid containing doping genes copyDNA** | **EJ1** | **EJ2** | **EJ3** | **EJ4** |
| --- | --- | --- | --- | --- | --- |
| ***GH2*** | 0.1 | 255 | 196 | 18 | 176 |
| ***GH2*** | 0.01 | 3 | 3 | 0 | 0 |
| ***IGF1*** | 0.1 | 282 | 558 | 835 | - |
| ***IGF1*** | 0.01 | 27 | 47 | 69 | - |
| ***IGF2*** | 0.1 | 120 | 110 | 57 | - |
| ***IGF2**** | 0.01 | 0 | 0 | 0 | - |

*GH2*-EJ1 = exon-exon junction 1 = *GH2*-003_ENST00000332800_ENSE00002552221_ENSE00002457996

*GH2*-EJ2 = exon-exon junction 2 = *GH2*-003_ENST00000332800_ENSE00002457996_ENSE00002225272

*GH2*-EJ3 = exon-exon junction 3 = *GH2*-003_ENST00000332800_ENSE00002225272_ENSE00002563835

*GH2*-EJ4 = exon-exon junction 4 = *GH2*-001_ENST00000423893_ENSE00002443150-ENSE00003499926

*IGF1*-EJ1 = exon-exon junction 1 = *IGF1*-201_ENST00000456098_ENSE00001421605-ENSE00000498265

*IGF1*-EJ2 = exon-exon junction 2 = *IGF1*-201_ENST00000456098_ENSE00000498265-ENSE00001363278

*IGF1*-EJ3 = exon-exon junction 3 = *IGF1*-201_ENST00000456098_ENSE00001363278-ENSE00001513560

*IGF2*-EJ1 = exon-exon junction 1 = *IGF2*-001_ENST00000416167_ENSE00001640131-ENSE00003583447

*IGF2*-EJ2 = exon-exon junction 2 = *IGF2*-001_ENST00000416167_ENSE00003583447-ENSE00003495975

*IGF2*-EJ3 = exon-exon junction 3 = *IGF2*-001_ENST00000416167_ENSE00003495975-ENSE00001588587

* unexpected low value (also in table f)

**Table f:** *GH2*, *IGF1*, *IGF2* copyDNA detection using all unique sequence reads

| **Gene** | **EJ1** | | **EJ2** | | **EJ3** | | **EJ4** | |
| --- | --- | --- | --- | --- | --- | --- | --- | --- |
| **%Plasmid** | **Total** | **I-E/E-I** | **Total** | **I-E/E-I** | **Total** | **I-E/E-I** | **Total** | **I-E/E-I** |
| ***GH2* (0.1)** | 13737 | 1923 | 18781 | 2629 | 20844 | 2918 | 21400 | 2996 |
| ***GH2* (0.01)** | 3464 | 2182 | 2310 | 1455 | 3015 | 1899 | 2878 | 1813 |
| ***IGF1* (0.1)** | 19050 | 857 | 24184 | 1088 | 16610 | 747 | - | - |
| ***IGF1* (0.01)** | 2143 | 536 | 2309 | 577 | 1878 | 469 | - | - |
| ***IGF2* (0.1)** | 5079 | 391 | 8383 | 645 | 8760 | 674 | - | - |
| ***IGF2* (0.01)*** | 844 | 388 | 1118 | 514 | 1191 | 548 | - | - |

*GH2*-EJ1 = exon-exon junction 1 = *GH2*-003_ENST00000332800_ENSE00002552221_ENSE00002457996

*GH2*-EJ2 = exon-exon junction 2 = *GH2*-003_ENST00000332800_ENSE00002457996_ENSE00002225272

*GH2*-EJ3 = exon-exon junction 3 = *GH2*-003_ENST00000332800_ENSE00002225272_ENSE00002563835

*GH2*-EJ4 = exon-exon junction 4 = *GH2*-001_ENST00000423893_ENSE00002443150-ENSE00003499926

*IGF1*-EJ1 = exon-exon junction 1 = *IGF1*-201_ENST00000456098_ENSE00001421605-ENSE00000498265

*IGF1*-EJ2 = exon-exon junction 2 = *IGF1*-201_ENST00000456098_ENSE00000498265-ENSE00001363278

*IGF1*-EJ3 = exon-exon junction 3 = *IGF1*-201_ENST00000456098_ENSE00001363278-ENSE00001513560

*IGF2*-EJ1 = exon-exon junction 1 = *IGF2*-001_ENST00000416167_ENSE00001640131-ENSE00003583447

*IGF2*-EJ2 = exon-exon junction 2 = *IGF2*-001_ENST00000416167_ENSE00003583447-ENSE00003495975

*IGF2*-EJ3 = exon-exon junction 3 = *IGF2*-001_ENST00000416167_ENSE00003495975-ENSE00001588587

Total = total reads aligned

* unexpected low value (also in table e)

I-E/E-I = genomic DNA specific intron-exon or exon-intron sequences
